# FATS inhibits the Wnt pathway and induces apoptosis through degradation of MYH9 and enhances sensitivity to paclitaxel in breast cancer

**DOI:** 10.1038/s41419-024-07164-w

**Published:** 2024-11-16

**Authors:** Jin-Xuan Song, Yue Wang, Zhi-Peng Hua, Yue Huang, Lin-Fei Hu, Meng-Ran Tian, Li Qiu, Hong Liu, Jun Zhang

**Affiliations:** 1 Department of Breast Cancer, Tianjin Medical University Cancer Institute & Hospital, National Clinical Research Center for Cancer, Tianjin’s Clinical Research Center for Cancer, Tianjin, PR China; 2 Key Laboratory of Breast Cancer Prevention and Therapy, Tianjin Medical University, Ministry of Education, Tianjin, PR China; 3 Key Laboratory of Cancer Prevention and Therapy, Tianjin, PR China; 4 Department of Breast Surgery, Women and Children’s Hospital, School of Medicine, Xiamen University, Xiamen, Fujian PR China; 5 Department of Thyroid and Neck Tumor, Tianjin Medical University Cancer Institute & Hospital, National Clinical Research Center for Cancer, Tianjin’s Clinical Research Center for Cancer, Tianjin, PR China; 6 Department of Cancer Cell Biology, Tianjin’s Key Laboratory of Cancer Prevention and Therapy, National Clinical Research Center for Cancer, Tianjin Medical University Cancer Institute and Hospital, Tianjin, PR China

**Keywords:** Breast cancer, Tumour biomarkers, Predictive markers, Tumour-suppressor proteins, Chemotherapy

## Abstract

Breast cancer is one of the most prevalent and diverse malignancies, and, with global cases increasing, the need for biomarkers to inform individual sensitivity to chemotherapeutics has never been greater. Our retrospective clinical analysis predicted that the expression of the fragile site-associated tumor suppressor (FATS) gene was associated with the sensitivity of breast cancer to neoadjuvant chemotherapy with paclitaxel. In vitro experiments subsequently demonstrated that FATS significantly increased the inhibitory effects of paclitaxel on breast cancer cells’ migration, growth, and survival. An interaction screen revealed that FATS interacted with MYH9 and promoted its degradation via the ubiquitin-proteasome pathway, thereby downregulating Wnt signaling. By overexpressing FATS and MYH9, we demonstrated that FATS enhanced paclitaxel-induced apoptosis in breast cancer cells by degrading MYH9 to downregulate the Wnt pathway. We also demonstrated in a mouse xenograft model that FATS significantly increased the chemosensitivity of breast cancer cells to paclitaxel in vivo. This study presents a new mechanism by which FATS interacts with MYH9 to suppress the Wnt/β-catenin signaling pathway and induce apoptosis, thus enhancing the sensitivity of breast cancer cells to paclitaxel chemotherapy. The results also propose novel biomarkers for predicting breast cancer sensitivity to neoadjuvant chemotherapy with paclitaxel. Finally, we provide in vivo evidence that the combination of paclitaxel with IWR-1, a novel Wnt pathway inhibitor, synergistically suppresses breast cancer growth, laying the foundation for future trials with this drug combination. These results therefore provide a number of potential solutions for more precise treatment of patients with breast cancer in the future.

## Introduction

Breast cancer is the most prevalent malignant tumor among women, posing a serious threat to their health worldwide [1]. Although there has been significant progress in diagnosis and treatment in recent years, the pathogenesis of breast cancer remains complex and diverse, and treatment outcomes vary among individuals. The issue of resistance to first-line chemotherapeutic drugs in some patients is becoming more prevalent. Therefore, it is becoming increasingly important to identify biomarkers for predicting drug sensitivity in order to inform precise treatment for individual patients with breast cancer.

Paclitaxel is a well-established chemotherapeutic agent for breast cancer and is commonly used as a first-line drug in neoadjuvant treatment [2]. However, some patients do not respond to paclitaxel treatment or achieve complete remission with neoadjuvant chemotherapy. For those who are not sensitive to paclitaxel, treatment may not only fail to achieve the desired therapeutic effect but may also delay more effective treatment and cause unnecessary secondary damage due to the many side effects of this agent [3]. Screening for sensitivity to paclitaxel by detecting specific biomarkers is therefore crucial for achieving effective and precise treatment for patients with breast cancer.

The fragile site-associated tumor suppressor (FATS) gene is a typical fragile site gene with abundant AT repeats in the intron and expression regulatory regions. FATS, which is localized at 10q26.2, was identified by our group as the first tumor suppressor associated with DNA damage-induced tumors [4]. Fluorescence in situ hybridization (FISH) experiments confirmed that FATS is located at the fragile site FRA10F. The FATS gene was not only found to be absent in the DNA of patients with breast, ovarian, and lung cancer [5], but its mRNA expression was reduced or even absent in many cancer cells and tumor tissues compared to normal cells and paraneoplastic tissues, respectively. Moreover, cells co-transfected with FATS expression carriers displayed significant anti-tumor activities in vitro and in vivo. Important progress has been made in understanding the mechanism by which the FATS gene inhibits tumor development. It was found that expression of the FATS gene significantly induces the protein expression of p21, an important inhibitory factor in cell cycle regulation, causing G1 cell cycle arrest [6]. It suggested that FATS plays a crucial role in preserving the stability of the genome. Additionally, FATS is a distinct ubiquitin-conjugating enzyme that does not rely on other ubiquitin-conjugating enzymes [7]. Previous studies have demonstrated the independent value of FATS mRNA in predicting overall survival in patients with NSCLC receiving cisplatin chemotherapy [8]. It was subsequently confirmed that enhanced expression of FATS rendered NSCLC cells significantly more sensitive to cisplatin-induced apoptosis [9]. The expression level of FATS mRNA therefore provides a new biomarker for patients receiving cisplatin chemotherapy. Furthermore, a study on breast cancer demonstrated that patients with high FATS expression had a better prognosis than those with low FATS expression [10]. Multifactorial analysis showed that high FATS expression was an independent prognostic factor for patients with breast cancer treated with radiotherapy. Subsequent cellular assays confirmed that FATS regulated the sensitivity of breast cancer to radiotherapy [11]. Previous studies have also demonstrated that another fragile site-associated gene, WWOX, plays a crucial role in facilitating paclitaxel treatment of ovarian cancer by sensitizing tumor cells to paclitaxel through an ER stress-induced apoptosis mechanism [12, 13]. Given the multi-faceted tumor-suppressing roles of FATS described above, we speculated that FATS may also enhance chemosensitivity to paclitaxel in breast cancer. We therefore investigated FATS expression levels in breast cancer specimens and, on finding a correlation with response to treatment, subsequently investigated the mechanism by which FATS may sensitize breast cancer to paclitaxel. Our results provide potential biomarkers to allow more precise treatment of patients with breast cancer, as well as new potential combinatorial therapies that warrant further investigation.

## Results

### The expression of FATS is associated with the prognosis of neoadjuvant paclitaxel therapy for breast cancer

**Fig. 1:**
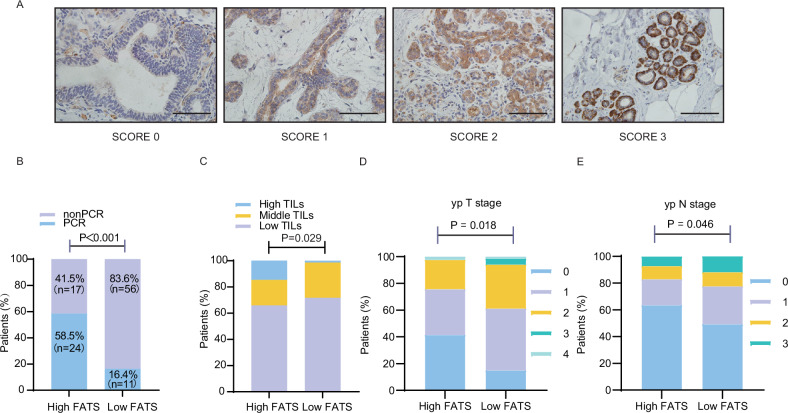
The clinicopathological data indicate that FATS expression is linked to sensitivity to neoadjuvant chemotherapy with paclitaxel in breast cancer. **A** Representative images of FATS IHC staining in crude needle-punctured breast tissue from 108 patients with triple-negative breast cancer are presented. The images were categorized into four types based on the intensity of staining: negative (score 0), weak (score 1), moderate (score 2), and strong (score 3). A scale bar of 100 μm is included. **B** The correlation between achieving pathological complete remission (pCR) status with neoadjuvant chemotherapy and FATS expression levels, using clinicopathological data. **C** The correlation between the percentage of tumor-infiltrating lymphocytes (TILs) and the level of FATS expression was analyzed using clinicopathological data. **D**, **E** The correlation between the yp TN stage and the expression level of FATS using clinicopathological data.

Based on the immunohistochemistry (IHC) score of the pathological tissue obtained from breast needle biopsy, 108 patients with breast cancer who underwent neoadjuvant chemotherapy were divided into two groups: those with high FATS expression and those with low FATS expression. Representative images of FATS IHC staining are shown in Fig. 1A. Information on the correlation between FATS protein expression and clinicopathological features in breast cancer patients receiving neoadjuvant therapy is demonstrated in Table 1. The study revealed that 58.5% of patients in the FATS high-expression group achieved postoperative pathologic complete response (pCR), accounting for 69% of the total pCR rate. In contrast, only 16.4% of patients in the FATS low-expression group achieved postoperative pCR, accounting for 31% of the total pCR rate (Fig. 1B). Table 2 shows the correlation between the achievement of pCR and clinicopathological features in breast cancer patients receiving neoadjuvant therapy with paclitaxel. Consistent with previous literature, significant associations were found between changes in ki67 (*p* = 0.006), ypTNM stage (*p* < 0.001), and AJCC stage (*p* < 0.001) with achieving pCR [14]. Table 2 illustrates the relationship between FATS expression and the clinicopathological characteristics of patients with breast cancer undergoing paclitaxel neoadjuvant chemotherapy. It was observed that high FATS expression correlates with increased androgen receptor expression (*p* = 0.019), tumor-infiltrating lymphocytes (*p* = 0.029), and ypTNM stage (*p* < 0.05) (Fig. 1C–E). The impact of ARs and TILs on the pCR of neoadjuvant chemotherapy remains elusive. Consequently, this study did not investigate whether FATS influences these factors to affect pCR. However, our experiments revealed that high FATS levels are positively correlated with postoperative pCR after breast cancer paclitaxel neoadjuvant chemotherapy (*p* < 0.001). This suggests that FATS could serve as a biomarker for predicting sensitivity to paclitaxel neoadjuvant chemotherapy in breast cancer.

**Table 1:** Correlation between FATS protein expression and clinicopathological features in patients undergoing neoadjuvant therapy for breast cancer.

Variables	Total (*n* = 108)	High expression (*n* = 41)	Low expression (*n* = 67)	*χ*^2^	*P*
PCR, *n* (%)				20.6	<0.001
nonPCR	73 (67.59)	17 (41.46)	56 (83.58)		
PCR	35 (32.41)	24 (58.54)	11 (16.42)		
Age groups, *n* (%)				2.73	0.17
≥51	58 (53.70)	16 (39.02)	42 (62.69)		
1–50	50 (46.30)	25 (60.98)	25 (37.31)		
Location of tumor, *n* (%)				0.09	0.769
L	56 (51.85)	22 (53.66)	34 (50.75)		
R	52 (48.15)	19 (46.34)	33 (49.25)		
Ki67 variations, *n* (%)				1.75	0.417
Unchanged	22 (20.37)	9 (21.95)	13 (19.40)		
Up	20 (18.52)	5 (12.20)	15 (22.39)		
Down	66 (61.11)	27 (65.85)	39 (58.21)		
P53, *n* (%)				0.07	0.784
H	65 (60.19)	24 (58.54)	41 (61.19)		
L	43 (39.81)	17 (41.46)	26 (38.81)		
CK5/6, *n* (%)				0.07	0.784
H	43 (39.81)	17 (41.46)	26 (38.81)		
L	65 (60.19)	24 (58.54)	41 (61.19)		
EGFR, *n* (%)				0.07	0.787
H	78 (72.22)	29 (70.73)	49 (73.13)		
L	30 (27.78)	12 (29.27)	18 (26.87)		
AR, *n* (%)				5.5	0.019
H	20 (18.52)	3 (7.32)	17 (25.37)		
L	88 (81.48)	38 (92.68)	50 (74.63)		
TILs groups, *n* (%)				-	0.029
H	7 (6.48)	6 (14.63)	1 (1.49)		
L	75 (69.44)	27 (65.85)	48 (71.64)		
M	26 (24.07)	8 (19.51)	18 (26.87)		
Histological Stage, *n* (%)				1.91	0.385
II	32 (29.63)	15 (36.59)	17 (25.37)		
II~III	27 (25.00)	8 (19.51)	19 (28.36)		
III	49 (45.37)	18 (43.90)	31 (46.27)		
Menopausal, *n* (%)				1.44	0.23
N	50 (46.30)	22 (53.66)	28 (41.79)		
Y	58 (53.70)	19 (46.34)	39 (58.21)		
NAC programmes, *n* (%)				-	0.492
TA	18 (16.67)	6 (14.63)	12 (17.91)		
TA + TE	2 (1.85)	1 (2.44)	1 (1.49)		
TAC	20 (18.52)	8 (19.51)	12 (17.91)		
TC	19 (17.59)	10 (24.39)	9 (13.43)		
TE	26 (24.07)	6 (14.63)	20 (29.85)		
TEC	20 (18.52)	9 (21.95)	11 (16.42)		
TEC + TAC	3 (2.78)	1 (2.44)	2 (2.99)		
yp T stage, *n* (%)				-	0.018
0	27 (25.00)	17 (41.46)	10 (14.93)		
1	45 (41.67)	14 (34.15)	31 (46.27)		
2	31 (28.70)	9 (21.95)	22 (32.84)		
3	3 (2.78)	0 (0.00)	3 (4.48)		
4	2 (1.85)	1 (2.44)	1 (1.49)		
yp N stage, *n* (%)				-	0.046
0	59 (54.63)	26 (63.41)	33 (49.25)		
1	27 (25.00)	8 (19.51)	19 (28.36)		
2	11 (10.19)	4 (9.76)	7 (10.45)		
3	11 (10.19)	3 (7.32)	8 (11.94)		
AJCC Stage, n(%)				-	0.045
0	23 (21.30)	15 (36.59)	8 (11.94)		
1A	24 (22.22)	7 (17.07)	17 (25.37)		
1B	1 (0.93)	1 (2.44)	0 (0.00)		
2A	28 (25.93)	9 (21.95)	19 (28.36)		
2B	9 (8.33)	1 (2.44)	8 (11.94)		
3A	10 (9.26)	4 (9.76)	6 (8.96)		
3B	2 (1.85)	1 (2.44)	1 (1.49)		
3C	11 (10.19)	3 (7.32)	8 (11.94)		

*χ*^2^ Chi-square test.

- Fisher exact.

**Table 2:** Correlation between the achievement of pathological complete remission and clinicopathological features in breast cancer patients receiving neoadjuvant therapy.

Variables	Total (*n* = 108)	nonPCR (*n* = 73)	PCR (*n* = 35)	*χ*^2^	*P*
FATS, *n* (%)				20.6	<0.001
H	41 (37.96)	17 (23.29)	24 (68.57)		
L	67 (62.04)	56 (76.71)	11 (31.43)		
Age groups, *n* (%)				0.11	0.743
≥51	58 (53.70)	40 (54.79)	18 (51.43)		
1–50	50 (46.30)	33 (45.21)	17 (48.57)		
Location of tumor, *n* (%)				0.58	0.446
L	56 (51.85)	36 (49.32)	20 (57.14)		
R	52 (48.15)	37 (50.68)	15 (42.86)		
Ki67 variations, *n* (%)				10.15	0.006
Unchanged	22 (20.37)	16 (21.92)	6 (17.14)		
Up	20 (18.52)	19 (26.03)	1 (2.86)		
Down	66 (61.11)	38 (52.05)	28 (80.00)		
P53, *n* (%)				1.66	0.198
H	65 (60.19)	47 (64.38)	18 (51.43)		
L	43 (39.81)	26 (35.62)	17 (48.57)		
CK5/6, *n* (%)				0.66	0.416
H	43 (39.81)	31 (42.47)	12 (34.29)		
L	65 (60.19)	42 (57.53)	23 (65.71)		
EGFR, *n* (%)				1.56	0.211
H	78 (72.22)	50 (68.49)	28 (80.00)		
L	30 (27.78)	23 (31.51)	7 (20.00)		
AR, *n* (%)				3.4	0.065
H	20 (18.52)	17 (23.29)	3 (8.57)		
L	88 (81.48)	56 (76.71)	32 (91.43)		
TILs groups, *n* (%)				-	0.3
H	7 (6.48)	3 (4.11)	4 (11.43)		
L	75 (69.44)	53 (72.60)	22 (62.86)		
M	26 (24.07)	17 (23.29)	9 (25.71)		
Histological stage, *n* (%)				5.22	0.074
II	32 (29.63)	19 (26.03)	13 (37.14)		
II~III	27 (25.00)	23 (31.51)	4 (11.43)		
III	49 (45.37)	31 (42.47)	18 (51.43)		
Menopausal, *n* (%)				0.01	0.933
N	50 (46.30)	34 (46.58)	16 (45.71)		
Y	58 (53.70)	39 (53.42)	19 (54.29)		
NAC programmes, *n* (%)				-	0.27
TA	18 (16.67)	12 (16.44)	6 (17.14)		
TA + TE	2 (1.85)	1 (1.37)	1 (2.86)		
TAC	20 (18.52)	14 (19.18)	6 (17.14)		
TC	19 (17.59)	8 (10.96)	11 (31.43)		
TE	26 (24.07)	23 (31.51)	3 (8.57)		
TEC	20 (18.52)	12 (16.44)	8 (22.86)		
TEC + TAC	3 (2.78)	3 (4.11)	0 (0.00)		
yp T stage, *n* (%)				-	<0.001
0	27 (25.00)	3 (4.11)	24 (68.57)		
1	45 (41.67)	38 (52.05)	7 (20.00)		
2	31 (28.70)	27 (36.99)	4 (11.43)		
3	3 (2.78)	3 (4.11)	0 (0.00)		
4	2 (1.85)	2 (2.74)	0 (0.00)		
yp N stage, *n* (%)				-	<0.001
0	59 (54.63)	29 (39.73)	30 (85.71)		
1	27 (25.00)	23 (31.51)	4 (11.43)		
2	11 (10.19)	11 (15.07)	0 (0.00)		
3	11 (10.19)	10 (13.70)	1 (2.86)		
AJCC stage, *n* (%)				-	<0.001
0	23 (21.30)	1 (1.37)	22 (62.86)		
1A	24 (22.22)	19 (26.03)	5 (14.29)		
1B	1 (0.93)	1 (1.37)	0 (0.00)		
2A	28 (25.93)	21 (28.77)	7 (20.00)		
2B	9 (8.33)	9 (12.33)	0 (0.00)		
3A	10 (9.26)	10 (13.70)	0 (0.00)		
3B	2 (1.85)	2 (2.74)	0 (0.00)		
3C	11 (10.19)	10 (13.70)	1 (2.86)		

*χ*^2^ Chi-square test.

- Fisher exact.

### FATS improves chemosensitivity to paclitaxel in breast cancer by enhancing apoptosis in vitro

**Fig. 2:**
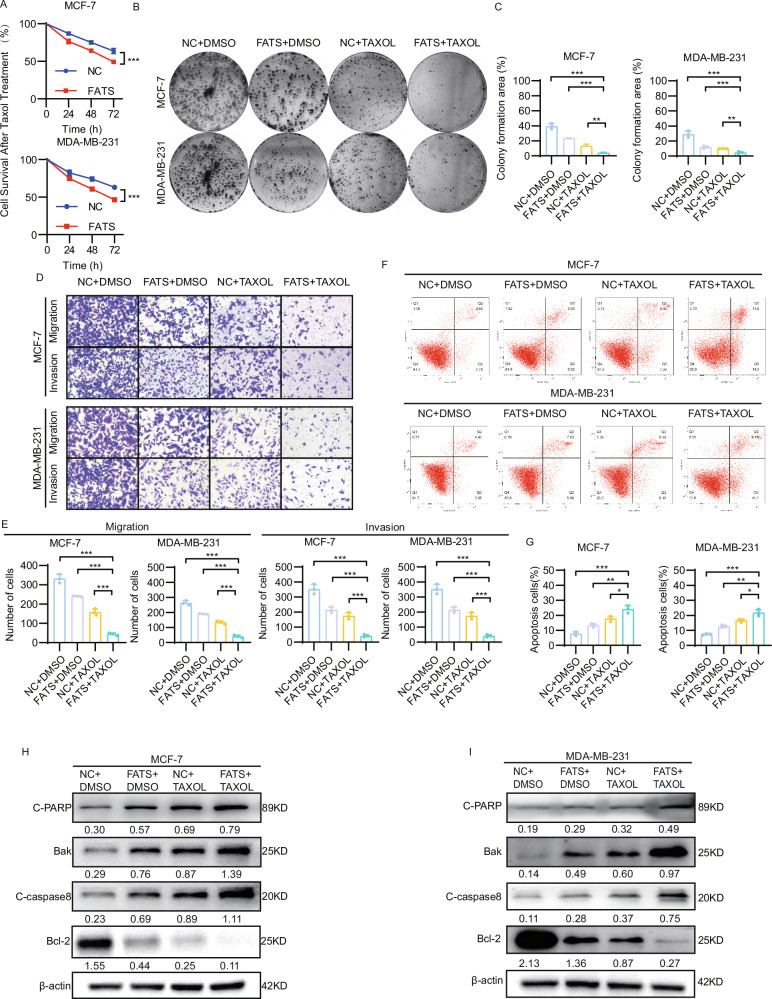
FATS works together with paclitaxel to enhance apoptosis in vitro, thereby improving chemosensitivity in breast cancer. **A** The CCK-8 proliferation assay was performed on MCF-7 and MDA-MB-231 cells that were transfected with either the FATS overexpression plasmid or the empty plasmid and then treated with a specific concentration of paclitaxel. **B**, **C** Clone formation assays were performed on MCF-7 and MDA-MB-231 cells that were transfected with either the FATS overexpression plasmid or the empty plasmid and given either DMSO or Taxol treatment. **D**, **E** For the transwell migration and invasion assays, DMSO or Taxol treatment was given to MCF-7 and MDA-MB-231 cells that were transfected with either the FATS overexpression plasmid or the empty plasmid. **F**, **G** Apoptosis detection assays were performed by flow cytometry on MCF-7 and MDA-MB-231 cells that were transfected with either the FATS overexpression plasmid or the empty plasmid and then treated with DMSO or Taxol. **H**, **I** Apoptosis-related indicators C-PARP, Bcl-2, Bak, and C-caspase8 were detected using western blot analysis in MCF-7 and MDA-MB-231 cells transfected with FATS overexpressing or empty plasmids. Gray value analysis was also performed. All data are shown as the mean ± SD; *P < 0.05; **P < 0.01; ***P < 0.001.

To investigate the impact of FATS on the efficacy of paclitaxel in breast cancer, we chose to use MCF-7 and MDA-MB-231 cells for FATS overexpression experiments. After treating both cell lines with equal concentrations of paclitaxel, it was found that the survival rate of cells in the FATS overexpression group was significantly lower than that of the control group over time (Fig. 2A). After paclitaxel treatment, the clone formation (Fig. 2B, C), migration, and invasion ability (Fig. 2D, E) of MCF-7 and MDA-MB-231 cells were significantly reduced upon overexpression of FATS, while significantly enhancing tumor cell apoptosis (Fig. 2F, G). We next analyzed the expression of three pro-apoptotic proteins (C-PARP, Bak, and C-caspase8) and an anti-apoptotic protein (Bcl-2) using western blotting (Fig. 2H, I). The findings indicated a marked increase in apoptotic flux in the group treated with the combination of FATS overexpression and paclitaxel in comparison to the groups treated with each intervention alone.

### FATS downregulates the Wnt pathway and interacts with MYH9

**Fig. 3:**
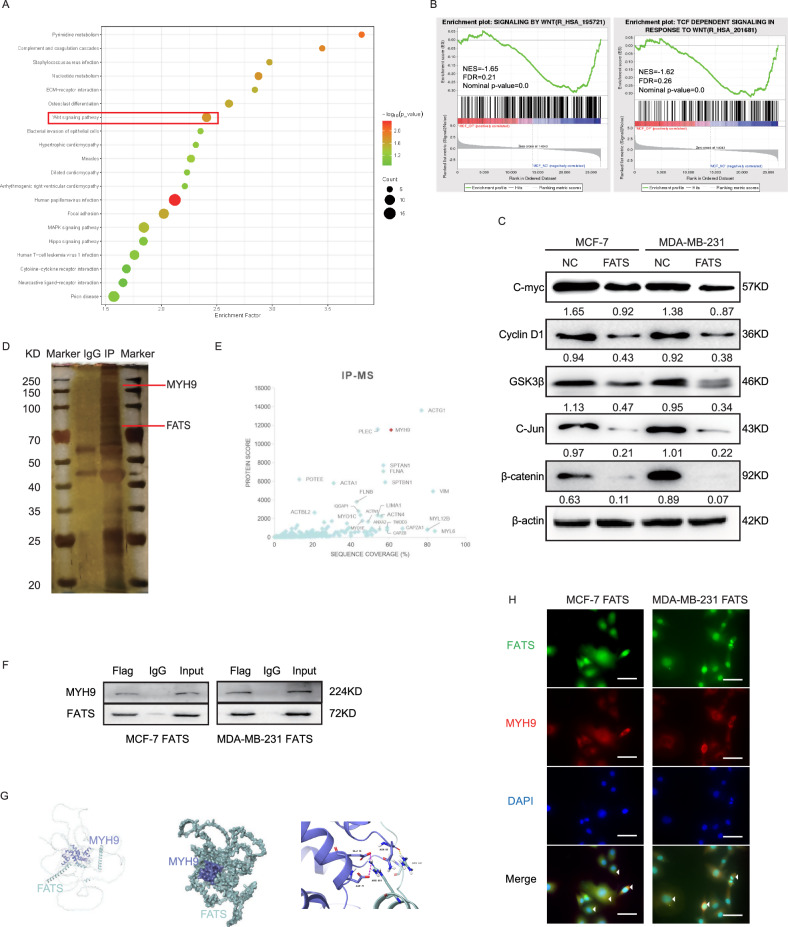
FATS downregulates the Wnt signaling pathway and binds to MYH9. **A**, **B** RNA-seq was performed on MCF-7 cells transfected with FATS overexpressing or empty plasmids. The KEGG analysis revealed that overexpression of FATS could affect the Wnt signaling pathway. Additionally, the GSEA analysis indicated a negative correlation between FATS expression and the set of genes associated with the Wnt signaling pathway in breast cancer cells. **C** Western blot detection of Wnt signaling pathway-related indicators β-catenin, C-myc, Cyclin D1, GSK3β, and C-Jun in MCF-7 cells and MDA-MB-231 cells transfected with FATS overexpression or null plasmid was performed and analyzed for gray scale values. **D** Co-immunoprecipitation (Co-IP) and silver staining were used to investigate the interaction between FATS and multiple proteins in MCF-7 cells transfected with Flag-FATS or an empty plasmid. **E** Following mass spectrometry analysis of the co-immunoprecipitated proteins, the differential proteins were presented based on their protein scores and coverage through scatter plots. **F** Binding of FATS and MYH9 was detected by Co-IP on MCF-7 and MDA-MB-231 cells transfected with Flag-FATS overexpressing or empty plasmids. **G** The AutoDock software was used to predict the amino acid sites involved in the interaction between FATS and MYH9. **H** Immunofluorescence analysis was used to detect co-localization of FATS and MYH9 in MCF-7 and MDA-MB-231 cells overexpressing FATS. FATS was labeled in green, MYH9 in red, and DAPI in blue. A scale bar of 20 μm is included.

To investigate the mechanism through which FATS improves the sensitivity of paclitaxel chemotherapy, we conducted RNA sequencing on breast cancer cells that overexpress FATS compared to their control cells. The dataset obtained from RNA sequencing was utilized to carry out Kyoto Encyclopedia of Genes and Genomes (KEGG) and Gene Set Enrichment Analysis (GSEA) [15–17], both of which suggested that FATS overexpression is significantly related to Wnt-associated pathways (Fig. 3A, B). Subsequently, we examined the expression of proteins downstream of the Wnt signaling pathway via western blotting. This revealed that the expression of β-catenin, C-myc, Cyclin D1, and GSK3β was reduced following FATS overexpression (Fig. 3C). We next performed immunoprecipitation and mass spectrometry experiments to identify proteins that interact with FATS. By analysing protein scores and coverage (Fig. 3 D, E) and retrieving whether these proteins were reported to regulate the Wnt signalling pathway, we identified that the top-ranked protein overall, actin gamma 1 (ACTG1), despite its capacity to bind to FATS (Fig. S1B) and having been previously reported to be Given its involvement in a wide range of cancers, it was not possible to ascertain whether this protein could affect the Wnt/β-catenin signaling pathway in breast cancer cells (Fig. S1C, D). Consequently, the second-ranked protein, myosin heavy chain 9 (MYH9), was selected for further investigation, given the extensive literature reporting its relationship with the Wnt pathway. A potential candidate for binding, MYH9, was identified by protein scoring and coverage (Fig. 3E, F). Co-immunoprecipitation experiments in both cell lines demonstrated that FATS interacts with MYH9 (Fig. 3F). Virtual molecular docking of the two proteins using AutoDock 4.2 software predicted possible binding sites (Fig. 3G). We also performed immunofluorescence experiments and found endogenous co-localization of FATS and MYH9 in the cytoplasm of breast cancer cells (Fig. 3H).

### FATS promotes the degradation of MYH9 by enhancing its ubiquitination

**Fig. 4:**
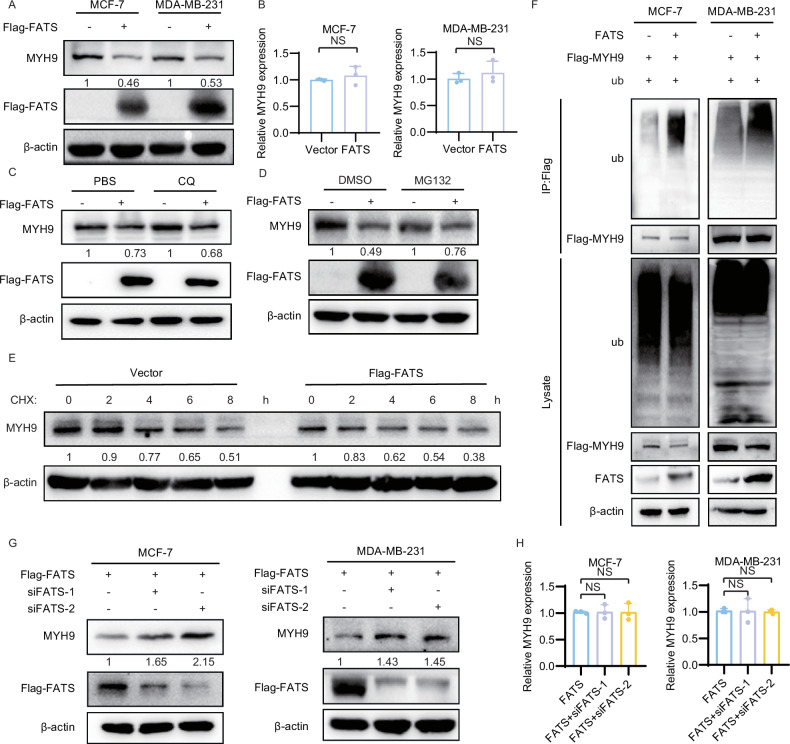
FATS promotes the degradation of MYH9 by enhancing its ubiquitination. **A** Protein levels of MYH9 in MCF-7 and MDA-MB-231 cells transfected with Flag-FATS overexpressing or empty plasmids were subjected to western blot detection and gray level analysis. **B** The mRNA levels of MYH9 were measured in MCF-7 and MDA-MB-231 cells that were transfected with either Flag-FATS overexpressing plasmids or empty plasmids. **C**, **D** The expression of MYH9 protein was detected and protein blotting gray values were calculated in MCF-7 cells overexpressing Flag-FATS or transfected with an empty plasmid, after treatment with MG132 and CQ. **E** Levels of MYH9 protein and gray scale analysis were conducted in CHX-treated MCF-7 cells expressing Flag-FATS or empty plasmids. **F** MYH9 and the indicated proteins were detected exogenously by denaturing immunoprecipitation of MCF-7 and MDA-MB-231 cells transfected with the specified plasmids. **G** Protein levels and gray value analysis of MYH9 were conducted in MCF-7 and MDA-MB-231 cells transfected with siNC or siFATS. **H** The mRNA levels of MYH9 were measured in MCF-7 and MDA-MB-231 cells that were transfected with either siNC or siFATS. All data are shown as the mean ± SD; NS, not significant.

To investigate the interaction between FATS and MYH9, we transfected MCF-7 and MDA-MB-231 cells with a FATS overexpression plasmid. The results of RT-qPCR and western blot analyses showed that altering the expression of FATS did not affect the mRNA level of MYH9 (Fig. 4B). However, overexpression of FATS led to a decrease in the protein level of MYH9 (Fig. 4A), while knockdown of FATS increased MYH9 levels (Fig. 4G, H). To investigate whether this was due to an effect on MYH9 stability, MDA-MB-231 cells were treated with CHX to inhibit protein synthesis. Assessment of MYH9 stability by western blotting indicated that the overexpression of FATS led to the destabilization of endogenous MYH9 protein and facilitated its degradation (Fig. 4E). To determine whether the degradation of MYH9 protein by FATS was achieved through the ubiquitin-proteasome or lysosomal pathway, we transfected FATS overexpression plasmids or empty vectors into MDA-MB-231 cells. The cells were then treated with either the proteasome inhibitor MG132 or the lysosomal inhibitor CQ (Fig. 4C, D). The study found that MYH9 degradation, which was mediated by FATS, was reversed by MG132 but not by CQ. This suggests that FATS promotes MYH9 degradation in a ubiquitin-proteasome-dependent manner. Additionally, we examined the effect of FATS on MYH9 ubiquitination by performing denaturing immunoprecipitations in both cell lines. The results showed that overexpression of FATS significantly increased exogenous polyubiquitination of MYH9 (Fig. 4F). Taken together, these results indicate that FATS facilitates the polyubiquitination of MYH9, resulting in its degradation through the ubiquitin-proteasome pathway.

### The sensitivity of FATS to paclitaxel chemotherapy in breast cancer is reversed by overexpression of MYH9

**Fig. 5:**
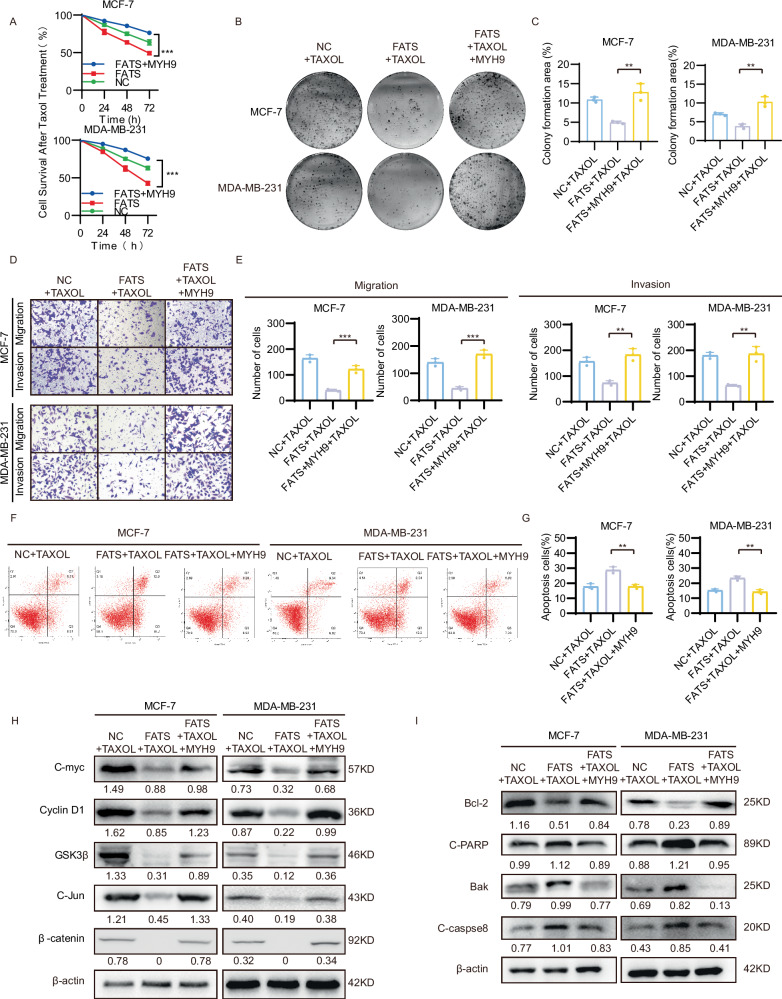
The overexpression of MYH9 negates the synergistic effects of FATS and paclitaxel in inhibiting the proliferation, migration, invasion, and apoptosis of breast cancer cells. **A** CCK-8 proliferation assay of MCF-7 and MDA-MB-231 cells transfected with the above plasmids under Taxol treatment. **B**, **C** Clone formation assay of MCF-7 and MDA-MB-231 cells transfected with the above plasmids under Taxol treatment. **D**, **E** Transwell migration and invasion assays of MCF-7 and MDA-MB-231 cells transfected with the above plasmids under Taxol treatment. **F**, **G** MCF-7 and MDA-MB-231 cells transfected with the above plasmids were treated with Taxol to detect apoptosis in each group using flow cytometry. **H**, **I** MCF-7 and MDA-MB-231 cells transfected with the above plasmids were treated with Taxol using western blot to detect the expression of Wnt pathway and apoptosis-related proteins and gray value analysis. All data are shown as the mean ± SD; **P < 0.01; ***P < 0.001.

To investigate whether FATS enhances the effect of paclitaxel on breast cancer cells by promoting the degradation of the MYH9 protein, we co-transfected FATS and MYH9 overexpression plasmids into MCF-7 and MDA-MB-231 cells. This demonstrated that the overexpression of FATS significantly enhanced the inhibitory effects of paclitaxel on the proliferation, migration, and invasion of breast cancer cells. Conversely, the overexpression of MYH9 restored the proliferation, migration, and invasion abilities of the cells (Fig. 5A–E). Nevertheless, our findings indicate that following MYH9 recovery, the outcomes of clone formation and migration were markedly superior to those observed in the NC group treated with paclitaxel alone. Consequently, we postulated that MYH9 might exert a role in breast cancer beyond that of FATS. To test this, we examined the proliferation, migration, and invasion of two cell lines following overexpression or knockdown of MYH9 (Fig. S2A, B). The results confirmed that MYH9 acts as an independent oncogenic factor, affecting the proliferation, migration, and invasion of breast cancer cells, thereby corroborating our hypothesis and aligning with previous literature [18, 19] (Fig. S2C–G). Subsequently, animal experiments were conducted, wherein the tumor size of the mouse breast cancer model with MYH9 knockdown was observed to be smaller than that of the control group. This finding was consistent with the results of the in vitro cellular experiments. Furthermore, both groups of mice were treated with paclitaxel, and it was observed that MYH9 knockdown led to an increased sensitivity to paclitaxel chemotherapy (Fig. S1H–K). Furthermore, we found that the overexpression of MYH9 reduced the promotion of apoptosis by FATS in synergy with paclitaxel, as detected by flow cytometry (Fig. 5F–G). Next, we detected the expression of Wnt pathway-related proteins and apoptosis-related proteins using western blotting. The results showed that overexpression of MYH9 reversed the reduction in the levels of the Wnt pathway-related proteins C-myc, Cyclin D1, GSK3β, C-Jun, and β-catenin caused by overexpression of FATS (Fig. 5H). MYH9 overexpression also rescued the decrease in the levels of pro-apoptosis-related proteins C-PARP, Bak, and C-caspase8 and the increase in the level of the anti-apoptotic protein Bcl-2 caused by FATS overexpression (Fig. 5I). The results of these rescue experiments suggest that FATS enhances chemosensitivity to breast cancer cells by degrading MYH9, downregulating the Wnt pathway, and promoting apoptosis (Fig. 7F).

### FATS overexpression and the Wnt pathway inhibitor IWR-1 both improve paclitaxel chemosensitivity in breast cancer in vivo

**Fig. 6:**
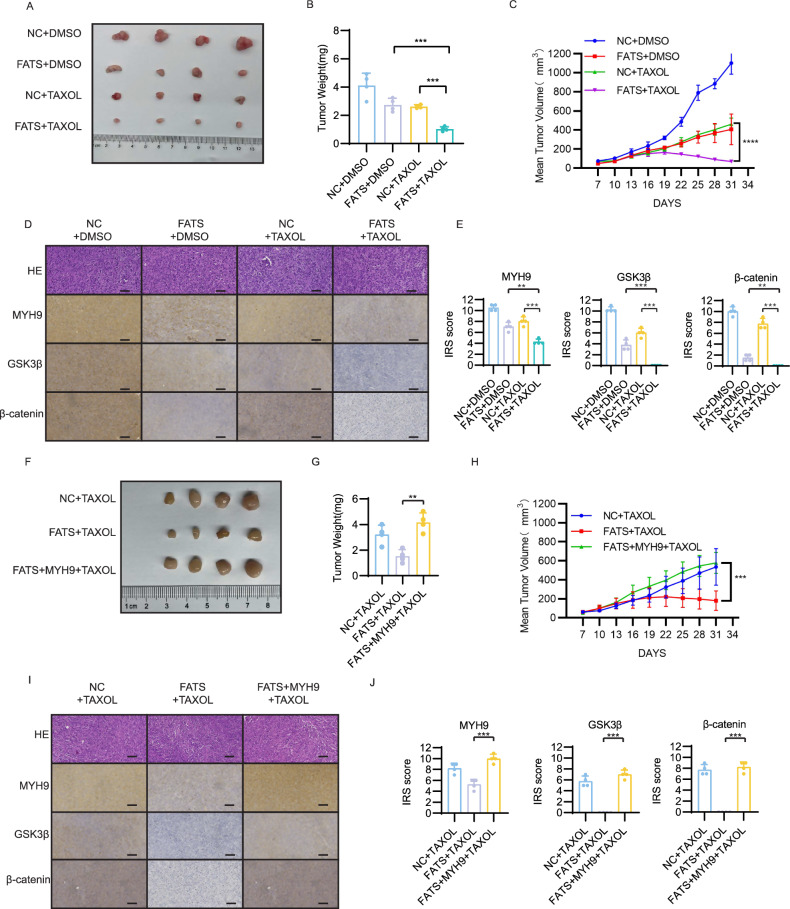
FATS improves paclitaxel chemosensitivity in breast cancer in vivo*.* **A** Representative images of tumors in a subcutaneous xenograft tumor model that overexpresses FATS or null plasmid after administration of Taxol and DMSO treatment, respectively. **B** Statistical comparison of tumor weights between the four groups. **C** Statistical comparison was conducted to analyze the growth trends of tumor volume in four groups of mice. **D** Representative images of four groups of tumor tissue H&E staining and immunohistochemical staining for MYH9, GSK3β, and β-catenin. Scale bar 50 μm. **E** Immunoreactive score statistics for MYH9, GSK3β, and β-catenin in four groups of tumor tissue. **F** Representative images of tumors overexpressing FATS or empty plasmid and tumors overexpressing FATS and MYH9 in a subcutaneous xenograft tumor model after Taxol treatment was given separately. **G** Statistical comparison of tumor weights between the three groups. **H** Statistical comparison was conducted to analyze the growth trends of tumor volume in three groups of mice. **I** Representative images of three groups of tumor tissue H&E staining and immunohistochemical staining for MYH9, GSK3β, and β-catenin. Scale bar 50 μm. **J** Immunoreactive score statistics for MYH9, GSK3β, and β-catenin in three groups of tumor tissue. All data are shown as the mean ± SD; **P < 0.01; ***P < 0.001; ****P < 0.0001.

To investigate whether FATS enhances paclitaxel chemosensitivity in vivo, we established a tumor model of breast cancer in mice. The FATS overexpression and paclitaxel combination group (FATS + TAXOL) showed significantly slower tumor growth and a trend toward tumor shrinkage in the later stages compared to the control plus paclitaxel group (NC + TAXOL). The FATS + TAXOL group had the smallest tumor volume and tumor weight after execution among the four groups (Fig. 6A–C). These observations may confirm that FATS acts synergistically with paclitaxel to kill breast cancer cells in vivo, consistent with our in vitro results. Subsequently, we aimed to confirm that in vivo, FATS enhances breast cancer paclitaxel chemosensitivity by degrading MYH9. We treated three groups of mouse breast cancer tumor models with paclitaxel: the first group (NC) did not overexpress FATS, the second group overexpressed FATS, and the third group overexpressed FATS and MYH9. The tumors in the third group were significantly larger than those in the second group (Fig. 6F–H). In vivo rescue experiments demonstrated that overexpression of MYH9 reduced the enhanced paclitaxel chemosensitivity due to FATS overexpression, aligning with the results of our in vitro cellular experiments. The immunohistochemical results of MYH9, GSK3β, and β-catenin in two animal experiments demonstrated that MYH9 levels were significantly decreased in the FATS overexpression group, and that the Wnt signaling pathway was markedly inhibited in combination with paclitaxel. Furthermore, the downregulation of the Wnt pathway induced by FATS could be reversed when overexpression of MYH9 was performed (Fig. 6D, E, I, J). The results of the immunohistochemical analysis conducted in the animal experiments are in accordance with the findings obtained in the cellular experiments.

**Fig. 7:**
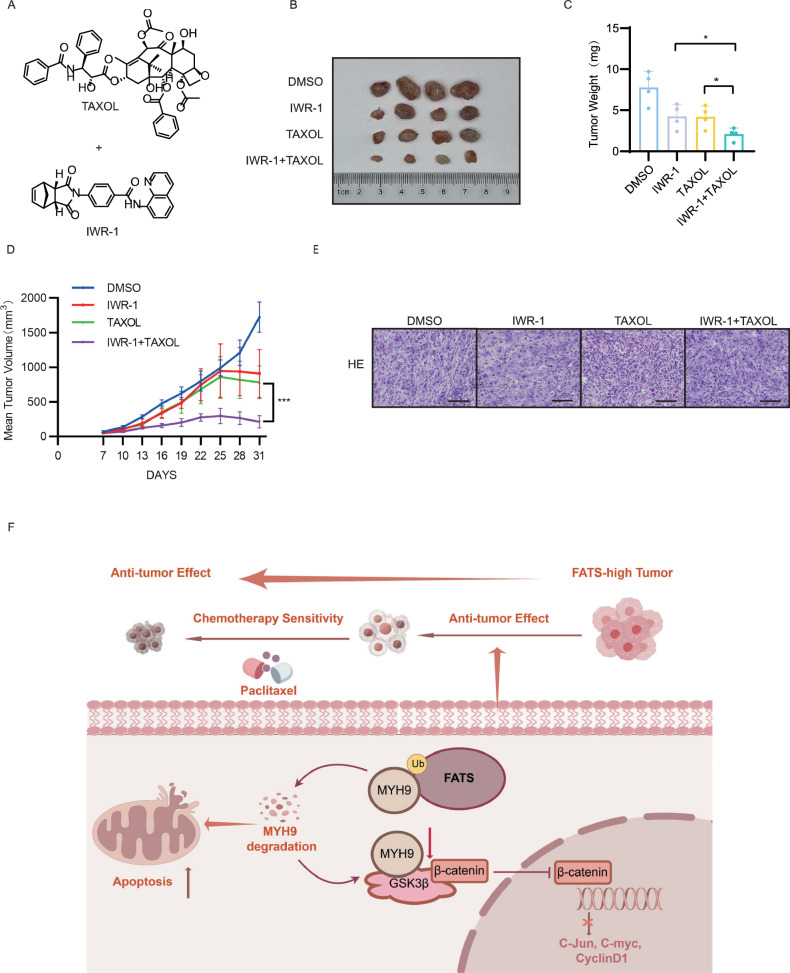
Wnt pathway inhibitor IWR-1 enhances breast tumor killing in combination with paclitaxel and mechanisms by which FATS enhances the sensitivity of breast cancer cells to paclitaxel chemotherapy. **A** Structural formulae of Taxol and IWR-1. **B** Representative images of a subcutaneous xenograft tumor model after treatment with Taxol and IWR-1 alone, as well as in combination, are presented. **C** Statistical comparison of tumor weights between the four groups. **D** Statistical comparison was conducted to analyze the growth trends of tumor volume in four groups of mice. **E** Representative images of a subcutaneous xenograft tumor model after treatment with Taxol and IWR-1 alone, as well as in combination, are presented. Scale bar 50 μm. **F** FATS has been demonstrated to downregulate GSK3β expression in the Wnt signaling pathway in breast cancer cells by recruiting MYH9 in the cytoplasm and promoting its degradation via the ubiquitin-proteasome pathway. This results in the degradation of β-catenin, which in turn leads to a reduction in the expression of C-myc, Cyclin D1, and C-Jun. Furthermore, FATS binds to MYH9 in the cytoplasm and promotes its degradation, thereby initiating apoptosis. All data are shown as the mean ± SD; *P < 0.05; ***P < 0.001.

As we have shown that FATS can sensitize paclitaxel by inhibiting the Wnt pathway through downregulation of β-catenin, we went on to utilize IWR-1, a Wnt pathway inhibitor that targets the β-catenin complex, in combination with paclitaxel in our breast cancer mouse model. We found that the combination of two drugs resulted in smaller tumor growth, volume, and weight compared to the group treated with paclitaxel or IWR-1 alone (Fig. 7A–E). This suggests that the Wnt pathway inhibitor IWR-1 can enhance the anti-tumor effects of paclitaxel in vivo.

## Discussion

Recent studies have shown that Wnt signaling is critical in regulating the immune microenvironment, maintaining stemness, shaping phenotype, and developing treatment resistance in breast cancer [20–24]. Fischer et al. found that blocking Wnt/β-catenin signaling prior to paclitaxel mitotic blockade effectively sensitized cancer stem cells (CSC) to paclitaxel, suggesting that modulation of the Wnt signaling pathway can influence paclitaxel chemosensitivity [25]. This is consistent with our findings that downregulation of the Wnt signaling pathway enhanced the sensitivity of breast cancer to paclitaxel chemotherapy.

MYH9 is known to promote tumor development, progression, and radiotherapy resistance [26]. For example, MYH9 is involved in glioma proliferation and drug resistance through the regulation of NAP1L1 deubiquitination [27]. Moreover, it has been shown that DNAJA4 inhibits invasion and metastasis of nasopharyngeal carcinoma through PSMD2-mediated proteasomal degradation of MYH9 [28]. MYH9 also interacts with GSK3β and reduces its protein expression through ubiquitin-mediated degradation, thereby dysregulating the β-catenin destruction complex and inhibiting cancer stemness and EMT [29–31]. In addition, SAMD9 promotes postoperative recurrence of esophageal squamous carcinoma through the MYH9/GSK3β/β-catenin axis [32]. The interaction between SMAD9 and MYH9 reduced MYH9 degradation by inhibiting activation of the ubiquitin-proteasome pathway and subsequent Wnt/β-catenin pathway. The promotion of gastric cancer metastasis is facilitated by MYH9-induced expression of β-catenin in the nucleus [33]. TUBB4A interacts with MYH9 in the nucleus. This interaction leads to a reduction in the activation of the associated epithelial–mesenchymal transition and promotes prostate cancer through GSK3β/β-catenin signaling [34]. Based on these findings, the mechanism by which MYH9 regulates the Wnt/GSK3β/β-catenin signaling pathway is relatively clear. In our preceding study, we discovered that the NH_2_-terminal structural domain of FATS exhibits ubiquitin ligase (E3) activity and assembles ubiquitin polymers via K11-, K29-, and K63-linkages, independently of the ubiquitin-coupling enzyme (E2) [7]. This indicates that the oncogene FATS is also capable of functioning in cells as an E3 ligase. Our experimental findings have revealed that the regulation of the Wnt signaling pathway by FATS is achieved by the ubiquitination of MYH9, which in turn serves as an E3 ligase. The regulation of the Wnt signaling pathway by FATS is achieved through MYH9, as we demonstrated experimentally. Additionally, our rescue experiments demonstrated that the mechanism by which FATS regulates breast cancer sensitivity to paclitaxel chemotherapy is also achieved through the MYH9/GSK3β/β-catenin axis.

Our aim was to demonstrate that FATS can serve as a biomarker for predicting the sensitivity of paclitaxel chemotherapy in breast cancer and to investigate the molecular mechanism of how it specifically improves chemosensitivity. Additionally, we aimed to apply the results of our basic experiments to clinical translation. There is evidence that the use of low-dose paclitaxel and the Wnt signaling inhibitor XAV939 may be helpful in the treatment of triple-negative breast cancer, as reported by Shetti et al. [35]. We identified another novel inhibitor targeting the Wnt/β-catenin pathway, IWR-1. Some studies have reported that IWR-1 inhibits osteosarcoma growth by eliminating CSC and also increases cancer cell sensitivity to adriamycin by blocking the efflux transporter mechanism in osteosarcoma resistance models [36, 37]. However, there have been no reports on whether IWR-1 has an inhibitory effect on breast cancer or whether it can promote paclitaxel sensitivity. Therefore, we combined conventional paclitaxel chemotherapy with IWR-1 in a breast cancer mouse model and demonstrated that IWR-1 can inhibit breast cancer growth and significantly improve the anti-tumor efficacy of paclitaxel in vivo. Our study suggests the potential for a second-line drug for patients with breast cancer who are insensitive to paclitaxel, laying the foundation for the future entry of IWR-1 into clinical trials.

Our study has various limitations. For example, there are several other potential interactors of FATS that could play a role in its anti-tumor activity. In the future, more in-depth research will be required to unravel the specific mechanisms of the FATS-Wnt pathway interaction, such as how upstream genes regulate FATS. Paclitaxel can inhibit the function of Tregs and reverse tumor immune escape. Therefore, combining paclitaxel with immunotherapy can improve therapeutic outcomes [38]. Clinically, paclitaxel combination therapy has been used to treat malignant tumors, including breast cancer, non-small cell lung cancer, and ovarian cancer. Previous studies have shown that deficiency in FATS significantly enhances macrophage-mediated innate immune responses and T-cell-mediated adaptive anti-tumor immune responses while suppressing immunosuppressive responses. This statement suggests that FATS may function similarly to immune checkpoints, indicating the potential application of FATS in cancer immunotherapy. An immunomodulator targeting FATS will be developed in combination with paclitaxel to test its improved chemosensitivity in vitro and in vivo. This could potentially provide a new avenue for precision treatment of breast cancer patients.

Taken together, our results showed that FATS downregulates the Wnt signaling pathway by interacting with MYH9 and promoting its degradation via the ubiquitin-proteasome pathway. In vitro, FATS significantly increased the inhibitory effects of paclitaxel on breast cancer cells, which were rescued by overexpressing MYH9. FATS also significantly increased the chemosensitivity of breast cancer cells to paclitaxel in vivo. Importantly, combining the Wnt inhibitor IWR-1 with paclitaxel had a significant synergistic anti-tumor effect in vivo. This study uncovers a new mechanism by which FATS interacts with MYH9 to counteract the Wnt pathway, thereby increasing the sensitivity of breast cancer cells to paclitaxel chemotherapy. Our findings also suggest new biomarkers for predicting breast cancer sensitivity to paclitaxel neoadjuvant chemotherapy.

## Materials and methods

### Patient and tissue sample collection

This study included 108 patients with breast cancer who underwent neoadjuvant therapy with paclitaxel at Tianjin Medical University Cancer Institute and Hospital from 2021 to 2023, with approval from the Ethics Committee of Tianjin Medical University Cancer Institute and Hospital. Tissue samples for analysis were collected during surgeries performed for clinical indications and were subjected to immunohistochemical staining after sectioning. The study was conducted in accordance with the principles of the Declaration of Helsinki and informed consent was obtained from all subjects. The number of subjects was based on the number of subjects in other clinical retrospective studies of neoadjuvant chemotherapy, as a means of ensuring a sufficient sample size.

### Immunohistochemical analysis

IHC was conducted on 4 µm paraffin-embedded tissue sections from patients with breast cancer and mouse tumors. Sections were deparaffinized, rehydrated, and heated in EDTA (pH 9.0). After blocking with 3% H_2_O_2_ followed by PBS washes, sections were incubated with primary antibodies overnight at 4 °C. The next day, after PBS washes, the sections were incubated with HRP-labeled secondary antibody for 50 min. DAB chromogenic reagent (ZSGB-Bio, Beijing, China) was applied according to the manufacturer’s instructions, followed by counterstaining with hematoxylin, xylene rinsing, and resin mounting. Images were captured with an Orthostatic fluorescence microscope and imaging system (OLYMPUS BX61, Japan).

### Cell culture and treatments

Human breast cancer cell lines MCF-7 and MDA-MB-231 were acquired from the American Type Culture Collection. These cells were cultured in DMEM, supplemented with 10% FBS (Gibco, Waltham, USA), and 1% penicillin–streptomycin (100 U/mL penicillin and 100 mg/mL streptomycin), in a 5% CO_2_ humidified atmosphere at 37 °C. Mouse breast cancer cell line PY8119 was acquired from the American Type Culture Collection. This cell was cultured in Ham’s F-12K, supplemented with 10% FBS (Gibco, Waltham, USA), and 1% penicillin–streptomycin (100 U/mL penicillin and 100 mg/mL streptomycin), in a 5% CO_2_ humidified atmosphere at 37 °C. Cells were treated with 10 μM MG132 (Selleck, TX, USA) or 50 μM chloroquine (CQ, Sigma) for 8 h for proteasome and lysosome inhibition, respectively. For transwell assays, cells were treated with 40 nM paclitaxel (GLPBIO, Montclair, USA) or 10 mM IWR-1 (Selleck, TX, USA). For protein synthesis inhibition, cells were treated with 100 μg/mL cycloheximide (CHX, Sigma) for various durations (0, 2, 4, 6, 8 h). Lipofectamine 3000 (Invitrogen, Carlsbad, CA, USA) was used to transfect cells with plasmid or small interfering RNA (siRNA; Hanbio, Shanghai, China) according to the instructions of the manufacturer. The siRNA sequences used in this study are listed in Supplementary Table 1.

### RNA isolation and RT-qPCR

Total RNA was extracted using TRIzol Reagent (Invitrogen, San Diego, CA, USA). For cDNA synthesis, 5 μg of RNA was reverse transcribed using M-MLV reverse transcriptase (ThermoFisher). RT-qPCR was conducted using SYBR Green I on the ViiA™ 7 Real-Time PCR Detection System (BioRad, Hercules, USA), following a specific thermal profile. Gene expression was calculated using the 2^−ΔΔCt^ method relative to β-actin. All experiments were performed independently in triplicate. PCR primers are listed in Supplementary Table 2.

### RNA-seq analysis

FATS overexpressing or control MCF-7 cells were lysed using an RNA extraction solution (G3013; Servicebio, China) and the mRNA was extracted according to the manufacturer’s protocol. The global gene expression profiles were determined by mRNA sequencing, which was performed at Novogene Technology (China). The raw RNA-Seq data were uploaded to the Sequence Read Archive under accession number PRJNA1094871.

### Western blot assay

The cells were washed with cold PBS, lysed, and proteins were extracted using RIPA buffer (Solarbio, Beijing, China) with added PMSF. Separation and extraction of cytosolic and cytoplasmic proteins were performed using the Cytosolic and Cytoplasmic Protein Extraction Kit (Beyotime, Shanghai, China). Protein concentrations were determined using a BCA Protein Assay Kit (Sangon Biotech, Shanghai, China). Equal quantities of protein (50 µg) were separated by SDS–PAGE and transferred to PVDF membranes (Roche, Basel, Switzerland). Membranes were blocked with 5% non-fat milk in TBST for 1 h, then incubated with primary antibodies overnight at 4 °C. This was followed by incubation with HRP-linked secondary antibodies and detection using Amersham ECL Plus Western Blotting Detection Reagent (GE Healthcare, Chicago, IL, USA). Quantification was performed using ImageJ where necessary. Antibodies are listed in Supplementary Table 3.

### Cell apoptosis assay

Cell apoptosis was detected using a FITC Annexin V Apoptosis Detection Kit I (Simubiotech, Tianjin, China), following the manufacturer’s protocol. Cells were first washed with cold PBS, then resuspended in binding buffer, and stained with Annexin V-APC and Propidium Iodide. Cell death was quantified using a FACSVerse flow cytometer (BD Bioscience, San Diego, CA, USA) and analyzed with FlowJo software. Gating strategies were applied to exclude debris and doublets. Cells were categorized as viable, early apoptotic, late apoptotic, or necrotic based on Annexin V-APC and PI staining.

### Immunoprecipitation and co-immunoprecipitation assays

For immunoprecipitation, cells were washed with PBS, lysed in RIPA buffer with inhibitor cocktail (Thermo, USA), and processed using protein A/G agarose (Thermo) following the manufacturer’s instructions. Lysates containing 1 mg of total protein were incubated overnight at 4 °C with magnetic beads conjugated to Flag, FATS, or control IgG antibodies. After washing, proteins were eluted and analyzed by western blotting. Immunoprecipitation of FATS and MYH9 was performed using a kit (Sangon Biotech, China) with 4 µg of anti-FATS or anti-IgG antibodies and anti-MYH9 antibodies for detection. For co-immunoprecipitation, after FATS and MYH9 were co-transfected into cells, the cells were processed as mentioned above before detection. At the end of gel electrophoresis, the results were visualized by western blotting and silver staining using a Fast Silver Stain Kit (Beyotime, Shanghai, China) according to the manufacturer’s instructions. Antibodies are listed in Supplementary Table 3.

### Immunofluorescence assay

Treated cells in 24-well plates were washed twice with pre-cooled PBS and fixed with 500 μl 4% paraformaldehyde per well for 20 min. After washing three times with pre-cooled PBS, the cells were permeabilized with 300 μl of 0.2% Triton X-100 per well with shaking for 15 min. After blocking with 5% BSA (Biosharp, China), the cells were incubated with specific primary antibodies overnight at 4 °C, washed, and then incubated with secondary antibodies for 1 h on a shaker, protected from light. Antibodies are listed in Supplementary Table 3. Finally, the coverslips were sealed with 3–5 μl of DAPI (Beyotime, Shanghai, China) and then observed with a confocal laser scanning microscope (Leica TCS SP5, Germany).

### Molecular docking assay

The PDB ID of MYH9 is 4ETO, and the UniProt ID of FATS is Q96M02. The process of molecular recognition was simulated on AutoDock 4.2 on a computer to find the optimal binding conformation of the protein and its ligand and to ensure that the overall binding free energy of the complex was minimized.

### Plasmids and cloning

Flag-FATS plasmid was constructed by in-frame insertion of full-length FATS cDNA into the p3xFlag-myc-CMV-26 vector (Sigma, Buchs, Switzerland). Flag-MYH9 plasmid was constructed by in-frame insertion of full-length MYH9 cDNA into the p3xFlag pLVX-NeoR vector (Hanbio, Shanghai, China). And shMYH9 plasmid was constructed by in-frame insertion of full-length shRNA into the pSLenti-U6-shRNA(MYH9) -CMV-EGFP-F2A-Puro-WPRE (OBIO, Shanghai, China).

### Cell viability and clonogenic assays

For cell viability, MCF-7 or MDA-MB-231 cells were seeded in 96-well plates (1000 cells/well). CCK-8 reagent (US Everbright, Suzhou, China) was added at a series of time points (0, 24, 48, 72 h) followed by incubation for 2 h at 37 °C. Absorbance at 450 nm was measured using a Synergy H1 Hybrid Multi-Mode Microplate Reader (BioTek). For clonogenic assays, cells were seeded in 6-well plates (1000 cells/well) and allowed to form colonies over 7–10 days. Colonies were fixed, stained, and counted.

### Transwell assay

The invasive and migratory capacity of MCF-7 and MDA-MB-231 cells was assessed using a transwell chamber (COSTAR, Suzhou, China). Cells were treated with various agents and seeded onto Matrigel-coated membranes. After 24 h, migrated cells were fixed, stained, and counted. This experiment was replicated three times.

### In vivo ubiquitination assay

Ubiquitin (Ub) was transfected into MCF-7 and MDA-MB-231 cells expressing vector or FATS. Cell lysates were prepared 24 h post-transfection and incubated with Ni-NTA beads (Qiagen, Venlo, The Netherlands) in binding buffer (20 mM Tris-HCl [pH 7.4], 150 mM NaCl, 0.1% Triton X-100, 5 mM EDTA, 2 mM imidazole) containing protease and phosphatase inhibitor cocktail (Thermo, USA). The beads were collected and washed with the binding buffer a total of four times, followed by analysis by western blotting.

### Tumor-xenografted mice model

The study protocol for BALB/c mice and C57BL/J mice was approved by the Animal Ethics Committee of Tianjin Medical University Cancer Institute and Hospital. Mice care and experimental procedures complied with national and international guidelines. Female BALB/c mice and C57BL/J mice (4 weeks old) were purchased from SPF (Beijing) biotechnology Co. Ltd. BALB/c mice were injected with control or MDA-MB-231 cells (1 × 10^6^ cells/0.1 mL PBS) overexpressing the corresponding plasmid, respectively, and were treated with 5 mg/kg paclitaxel or an equivalent amount of DMSO every other day for 2 weeks after tumor formation. 1 × 10^6^ PY8119 cells were suspended in 0.1 mL of PBS, mixed with Matrigel (REF354234, Corning, USA), and injected subcutaneously into C57BL/J mice. Post-tumor formation, C57BL/J mice were treated with 5 mg/kg paclitaxel (GLPBIO, Montclair, USA), 5 mg/kg IWR-1 (Selleck, TX, USA), or both agents combined. Body weight and tumor volume were monitored, and tumor tissues were collected for analysis and histological examination. The number of animals in all experimental groups was four, in accordance with the principles of animal welfare and based on experience gained from previous studies. All animals were randomly assigned to the groups, and the experiments were conducted in a single-blind method.

### Hematoxylin and eosin (H&E) staining

H&E staining was performed on paraffin-embedded tumor tissues from mice xenografted with MDA-MB-231. After deparaffinization and rehydration, sections were stained with hematoxylin, rinsed, treated with acidic ethanol, and stained with eosin. After dehydration, images were captured with an Orthostatic fluorescence microscope and imaging system (OLYMPUS BX61, Japan).

### Statistical analysis

Data normalization was performed relative to control values and expressed as mean ± SD. The Chi-squared, Fisher’s exact, and two-tailed Student’s *t*-tests were utilized to compare date groups as appropriate. GraphPad Prism 9.0 software facilitated both statistical analysis and graphical representation. Independent prognostic factors were identified using a multivariate Cox regression model. Statistical significance was established at *p* < 0.05: the specific values are reported or indicated in figure legends as *p < 0.05; **p < 0.01; ***p < 0.001; ****p *<* 0.0001.

## Supplementary information

Supplementary Figures and Tables

western blot raw data

## Data Availability

The datasets generated during and/or analyzed during the current study are available from the corresponding author on reasonable request.
