# Supplementary material for: FATS inhibits the Wnt pathway and induces apoptosis through degradation of MYH9 and enhances sensitivity to paclitaxel in breast cancer
Source: Cell Death Dis. 2024 Nov 16;15(11):835. doi: 10.1038/s41419-024-07164-w (PMC11569202; doi:10.1038/s41419-024-07164-w)
Supplement: Supplementary file 1 — Supplementary Figures and Tables [file 41419_2024_7164_MOESM1_ESM.docx]

**Supplementary Figures and Tables**

**FATS inhibits the Wnt pathway and induces apoptosis through degradation of MYH9 and enhances sensitivity to paclitaxel in breast cancer**

Jin-Xuan Song^123#^,Yue Wang^123#^,Zhi-Peng Hua^4#^,Yue Huang^35^,Lin-Fei Hu^35^,Meng-Ran Tian^35^,Li Qiu^6^,Hong Liu^123^,Jun Zhang^123^

Author information

1. Department of Breast Cancer, Tianjin Medical University Cancer Institute & Hospital,National Clinical Research Center for Cancer, Tianjin’s Clinical Research Center for Cancer, Tianjin 300060, P. R. China
2. Key Laboratory of Breast Cancer Prevention and Therapy, Tianjin Medical University,Ministry of Education, Tianjin 300060, P. R. China
3. Key Laboratory of Cancer Prevention and Therapy,Tianjin 300060, P. R. China

4.Department of Breast Surgery, Women and Children's Hospital, School of Medicine, Xiamen University.No.10, Zhenhai Road, Xiamen 361003,Fujian P.R. China

5.Department of Thyroid and Neck Tumor, Tianjin Medical University Cancer Institute & Hospital,National Clinical Research Center for Cancer, Tianjin’s Clinical Research Center for Cancer, Tianjin 300060, P. R. China

6.Department of Cancer Cell Biology, Tianjin’s Key Laboratory of Cancer Prevention and Therapy, National Clinical Research Center for Cancer, Tianjin Medical University Cancer Institute and Hospital, Tianjin 300060, P. R. China.

#These authors contributed equally Jin-Xuan Song,Yue Wang,Zhi-Peng Hua

Correspondence: Jun Zhang [(doctorjunzhang@163.com)、](mailto:(doctorjunzhang@163.com)、)

Hong [Liu (liuhong_submit@tjmuch.com)](mailto:Liu(liuhong_submit@tjmuch.com))、Li [Qiu(liqiu@tmu.edu.cn)](mailto:Qiu(liqiu@tmu.edu.cn))


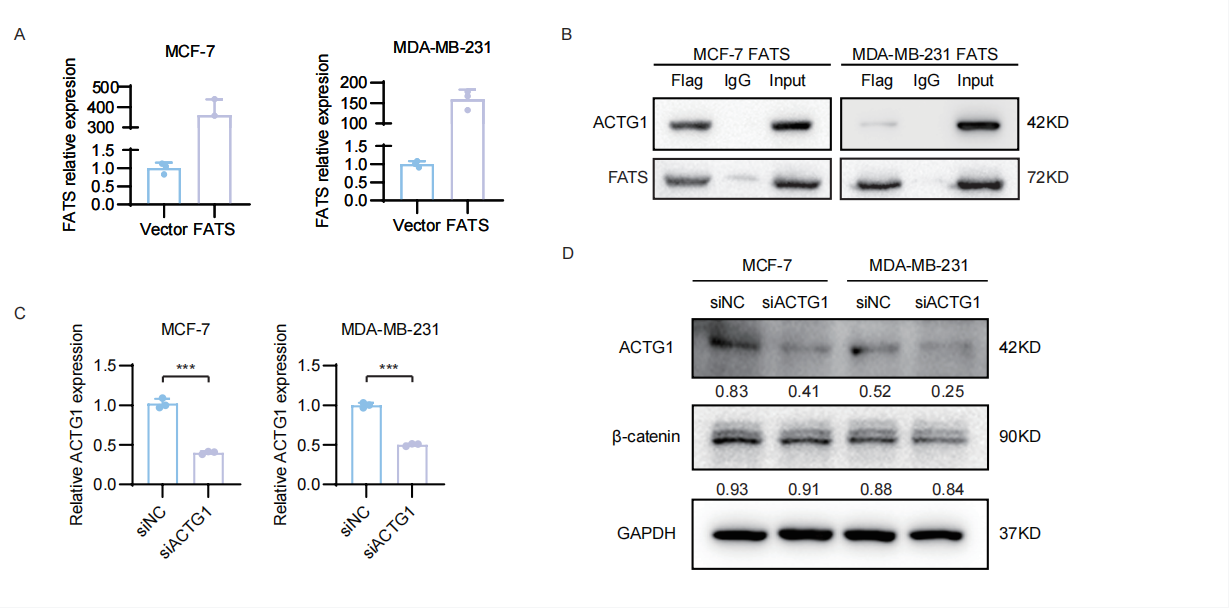


**FigS1 ACTG1 binds to FATS but does not affect wnt signalling pathway expression in breast cancer cells**

1. The mRNA levels of FATS were measured in MCF-7 and MDA-MB-231 cells that were transfected with either Flag-FATS overexpressing plasmids or empty plasmids.
2. Binding of FATS and ACTG1 was detected by Co-IP on MCF-7 and MDA-MB-231 cells transfected with Flag-FATS overexpressing or empty plasmids.
3. The mRNA levels of ACTG1 were measured in MCF-7 and MDA-MB-231 cells that were transfected with either siACTG1 or siNC.
4. Western blotting for ACTG1 and β-catenin was conducted in knockdown ACTG1 and control MCF-7 cells and MDA-MB-231 cells, and the grey values were subsequently analysed.


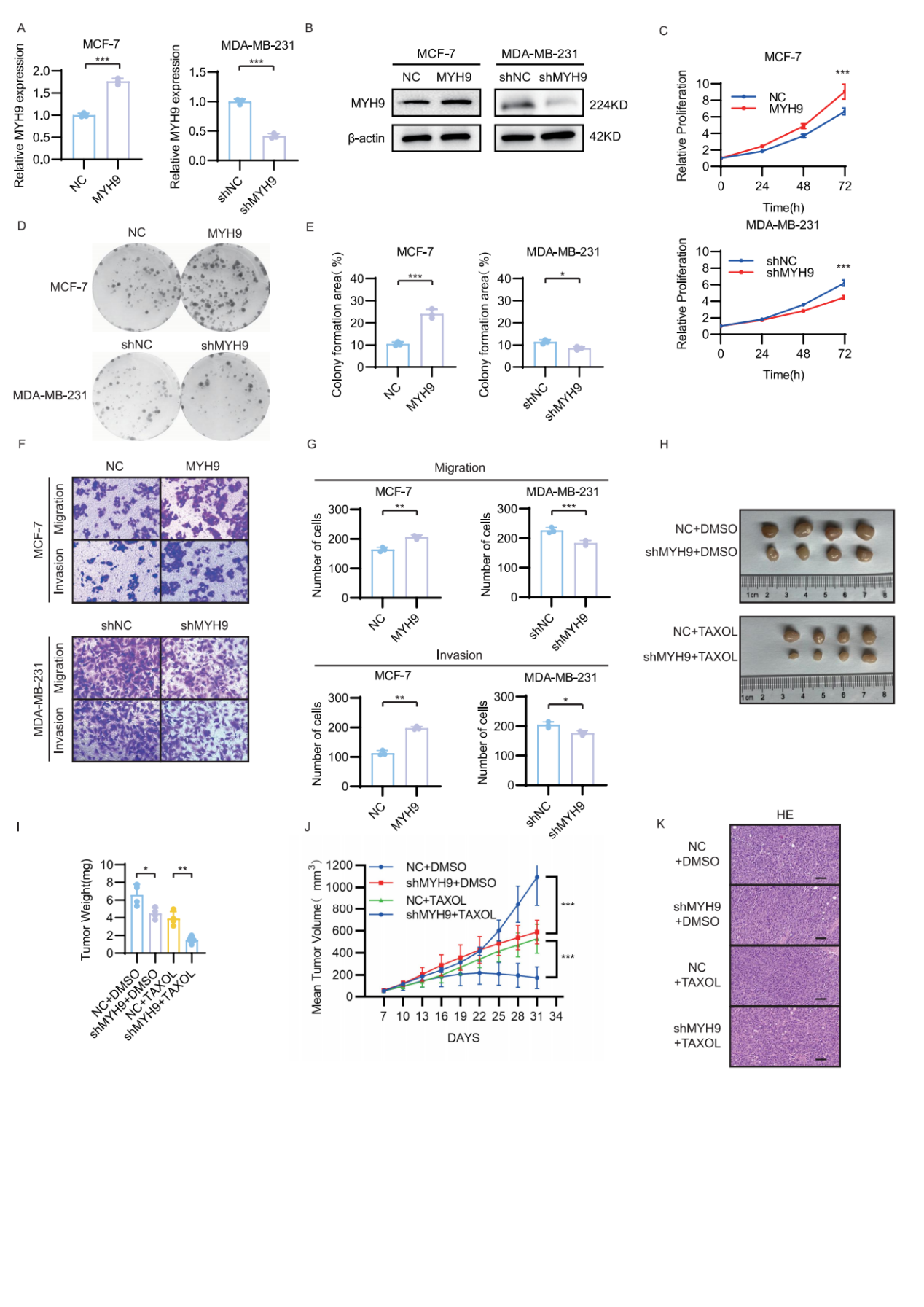


**FigS2 MYH9 affects breast cancer development *in vitro* and *in vivo***

A-B.Detection of protein and mRNA expression of MYH9 in MCF-7 cell line after overexpression of MYH9 and after knockdown of MYH9 in MDA-MB-231 cell line in both cells

C.CCK-8 experiments were performed on both of these cells

D-E.Clone formation experiments on two cell lines

F-G.The transwell migration and invasion assays on two cell lines

H.Representative images of a mouse breast cancer model constructed with knockdown MYH9 and control MDA-MB-231 cells with paclitaxel treatment

I.Statistical comparison of tumour weights between the four groups.

J.Statistical comparison was conducted to analyze the growth trends of tumour volume in four groups of mice.

K.Representative images of H&E staining of four groups of tumour tissues. Scale bar 50 μm.

Supplemental Table 1

siRNA information

| siFATS-1 |  | 5’-GAGATCAAATTGCCCTTAA-3’ |
| --- | --- | --- |
| siFATS-2 |  | 5’-CCACAGUAGAAGAGAUCAA-3’ |
| siACTG1 |  | 5’-GCUGGCAAGAACCAGUUGUUUTT-3’ |
| siNC |  | 5’-UUCUCCGAACGUGUCACGUTT-3’ |

Supplemental Table 2

RT-qPCR primer sequences

| FATS | Forward | 5’-TGTGCCATTGCTCAGTCTCG-3’ |
| --- | --- | --- |
|  | Reverse | 5’-TCTGTGAATGTTGACTCCGCT-3’ |
| MYH9 | Forward | 5’-CAGCAAGCTGCCGATAAGTAT-3’ |
|  | Reverse | 5’-CTTGTCGGAAGGCACCCAT-3’ |
| ACTG1 | Forward | 5’-CATTGTCATGGACTCTGGAGAC-3’ |
|  | Reverse | 5’-GAGGATCTTCATGAGGTAGTCG-3’ |
| β-actin | Forward | 5’-GGCTGTATTCCCCTCCATCG-3’ |
|  | Reverse | 5’-CCAGTTGGTAACAATGCCATGT-3’ |
| GAPDH | Forward | 5’-AGCCACATCGCTCAGACAC-3’ |
|  | Reverse | 5’-GCCCAATACGACCAAATCC-3’ |

Supplemental Table 3

Antibody list

| Antibody name | Product code and manufacturer | Antibody dilution in experiments |
| --- | --- | --- |
| FATS rabbit polyclonal | Cat. no. ab122497, Abcam, UK | WB 1:1000;IP 2.0 µg/ml; IF 1:200; IHC 1:100 |
| Flag mouse monoclonal | Cat. no. F1804, Sigma-Aldrich, USA | WB 1:5000; IP 1 µg/ml |
| Bak rabbit monoclonal | Cat. no. A5068, Selleck, USA | WB 1:1000 |
| Cyclin D1 rabbit monoclonal | Cat. no. A5035, Selleck, USA | WB 1:1000 |
| Cleaved PARP rabbit monoclonal | Cat. no. A5034, Selleck, USA | WB 1:1000 |
| MYH9 rabbit polyclonal | Cat. no.11128-1-AP, Proteintech, USA | WB 1:5000; IF 1:200 |
| MYH9 rabbit polyclonal | Cat. no. 51053-1-Ig, Proteintech, USA | IHC 1:200 |
| Beta Catenin rabbit polyclonal | Cat. no. 51067-2-AP, Proteintech, USA | WB 1:5000 |
| c-Myc rabbit monoclonal | Cat. no. 13987, Cell Signaling Technology, USA | WB 1:1000 |
| GSK-3β rabbit monoclonal | Cat. no. 12456, Cell Signaling Technology, USA | WB 1:1000 |
| c-Jun rabbit monoclonal | Cat. no. 9165, Cell Signaling Technology, USA | WB 1:1000 |
| β-Actin rabbit monoclonal | Cat. no. 4970, Cell Signaling Technology, USA | WB 1:2000 |
| Bcl-2 rabbit monoclonal | Cat. no. 3498, Cell Signaling Technology, USA | WB 1:1000 |
| Cleaved Caspase-8 rabbit monoclonal | Cat. no. 9496, Cell Signaling Technology, USA | WB 1:1000 |
| normal mouse IgG | Cat. no. sc-2025, Santa Cruz Biotechnology, USA | IP 2.0 µg/ml |

WB: Western blot; IHC: Immunohistochemistry; IF: Immunofluorescence; IP:Immunoprecipitation
