# Supplementary figures and images for: FATS inhibits the Wnt pathway and induces apoptosis through degradation of MYH9 and enhances sensitivity to paclitaxel in breast cancer
Source: Cell Death Dis. 2024 Nov 16;15(11):835. doi: 10.1038/s41419-024-07164-w (PMC11569202; doi:10.1038/s41419-024-07164-w)

Fig2

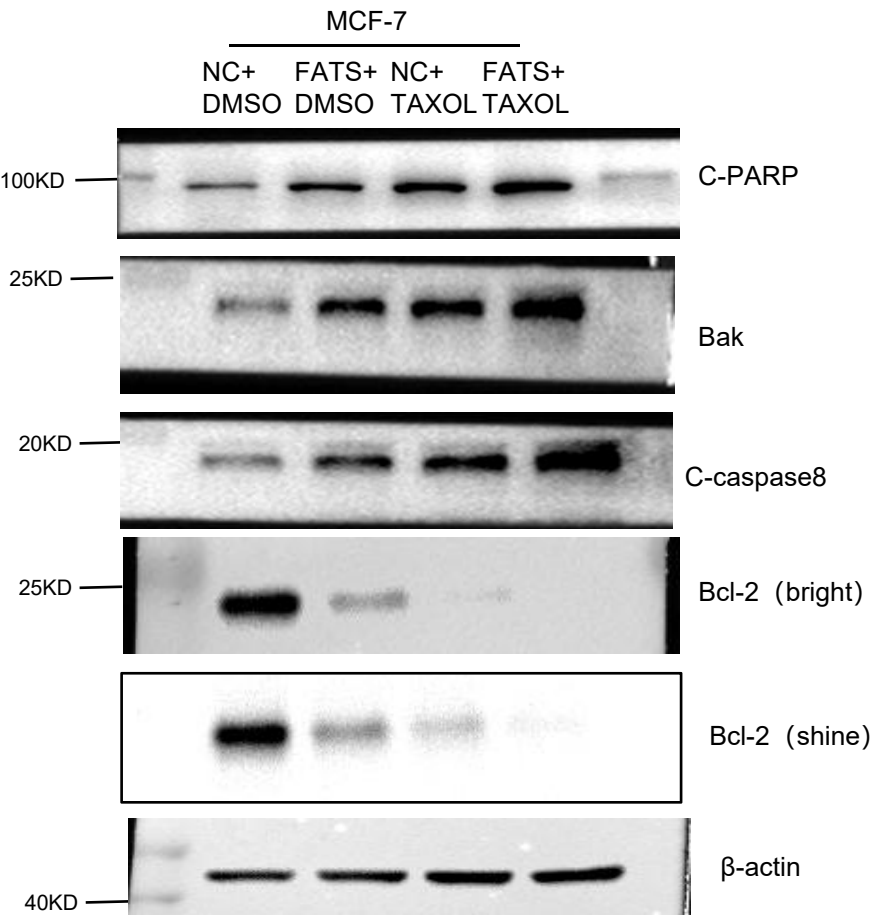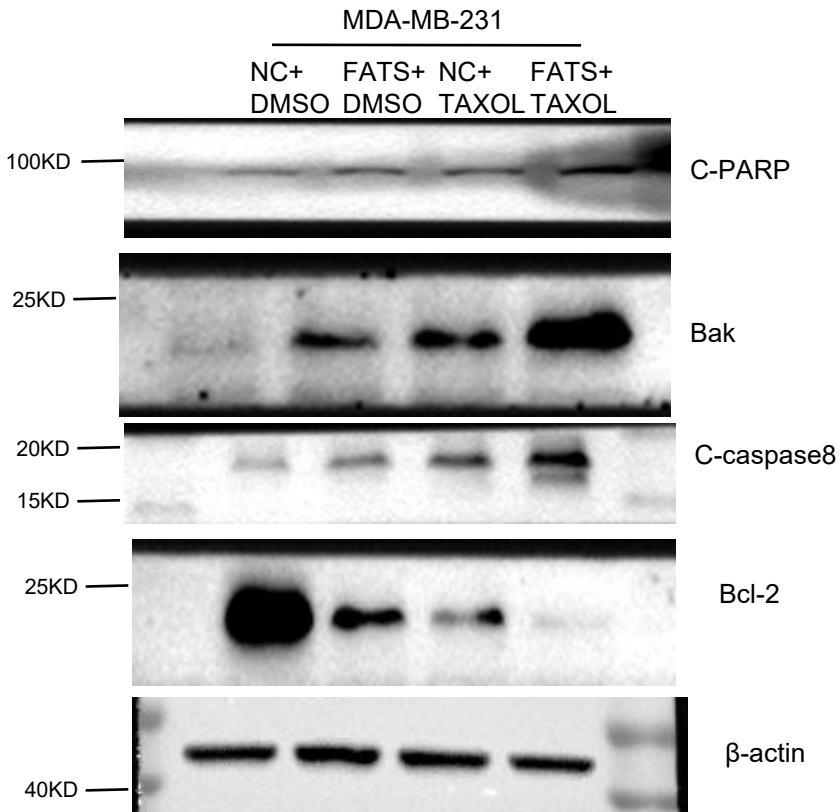

Fig3

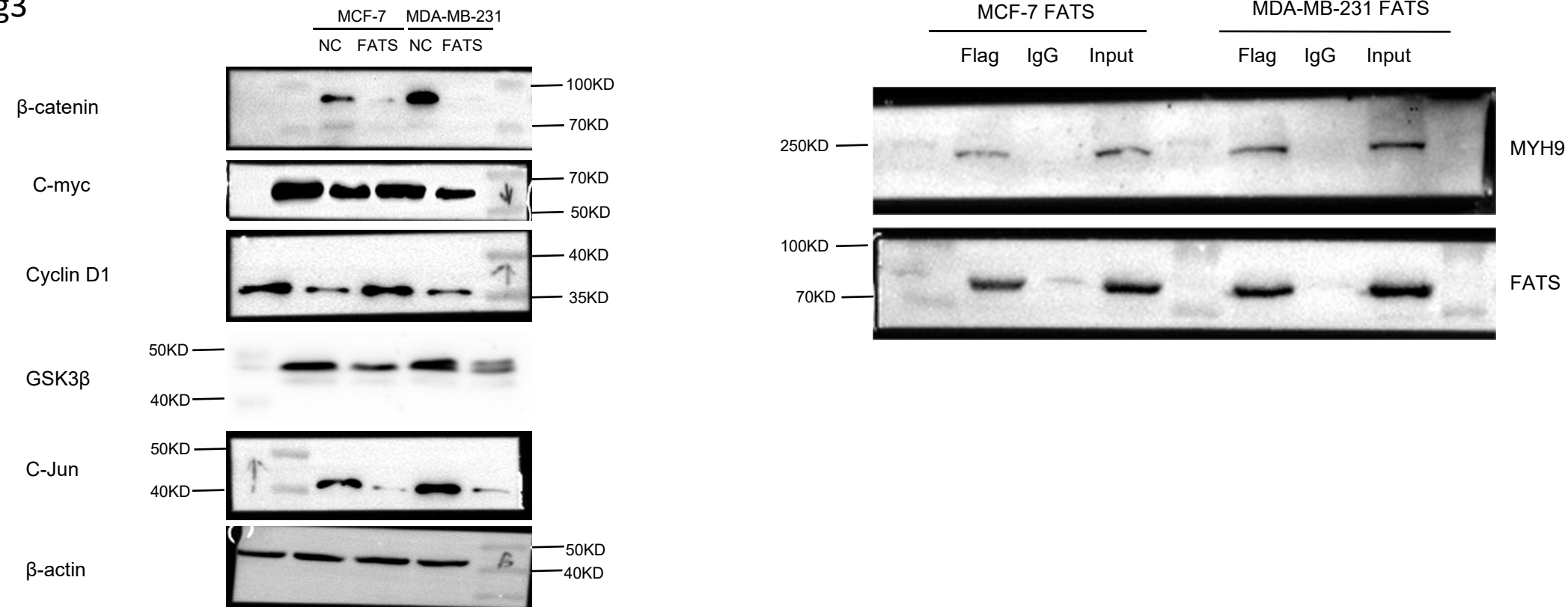

Fig4

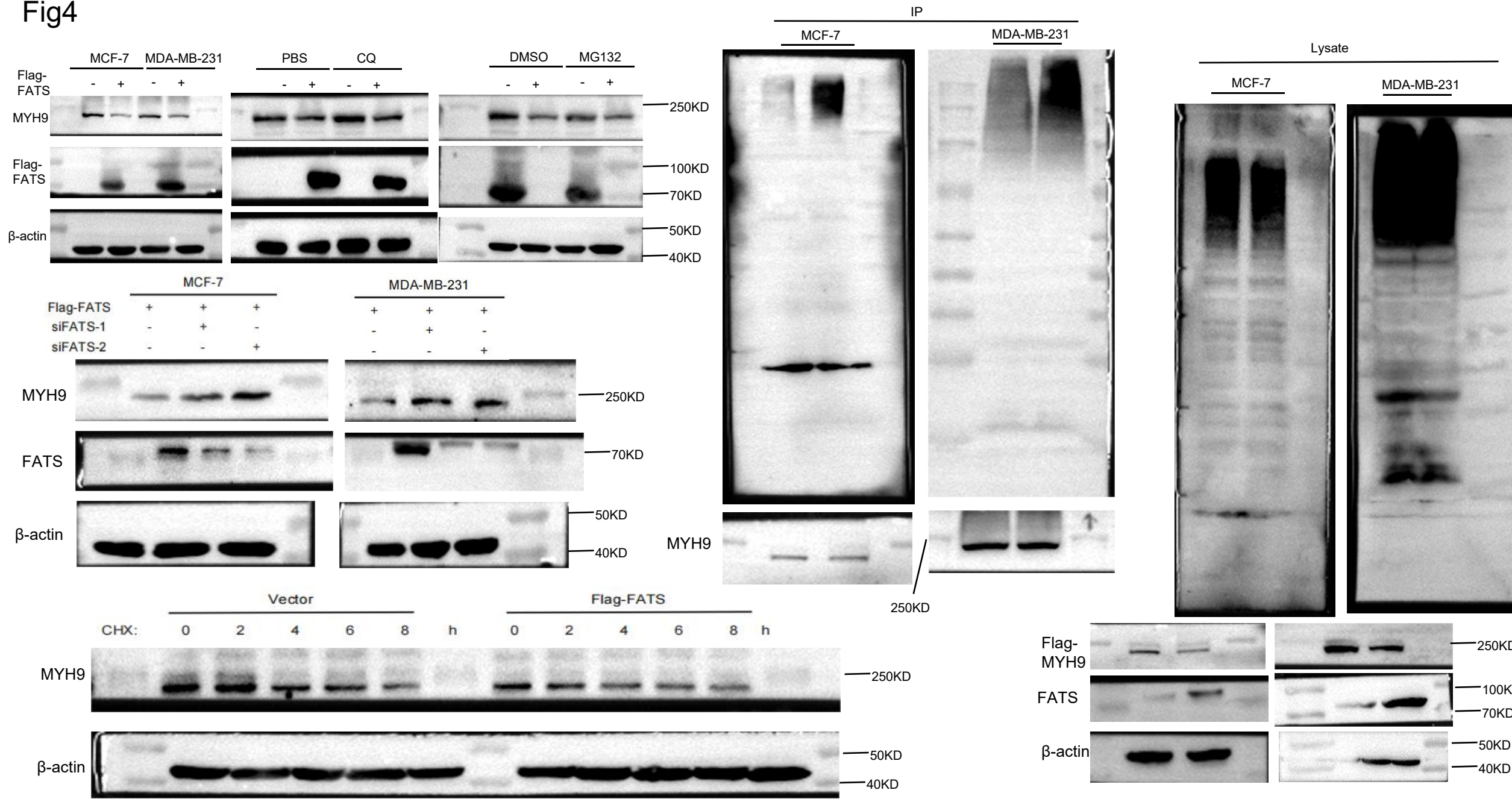

Fig5

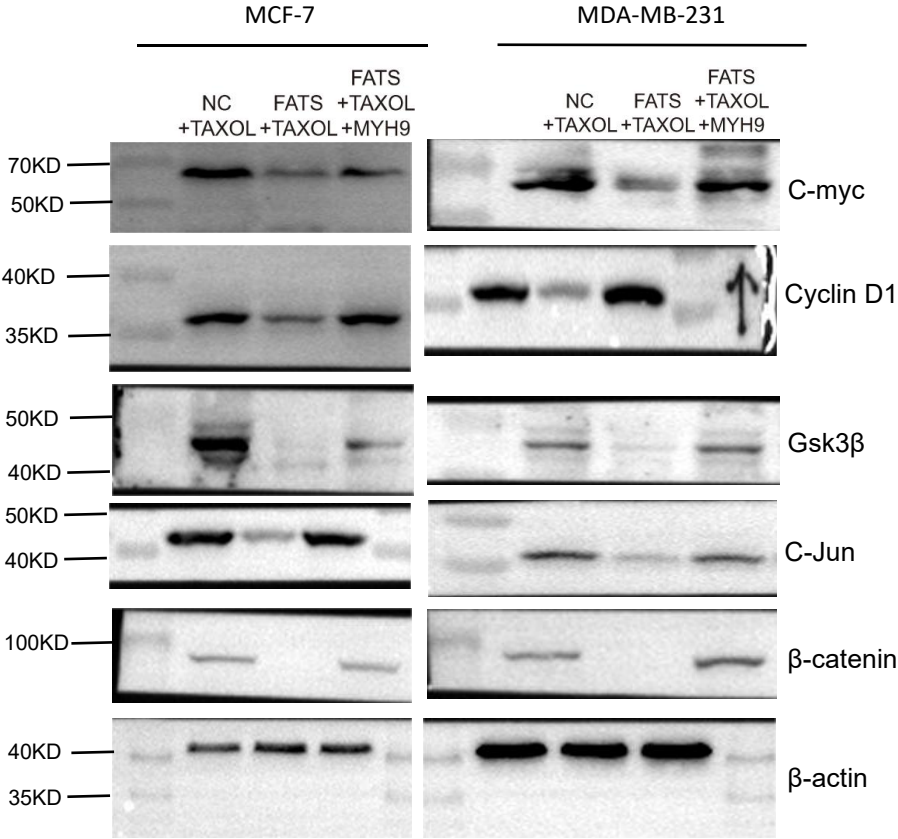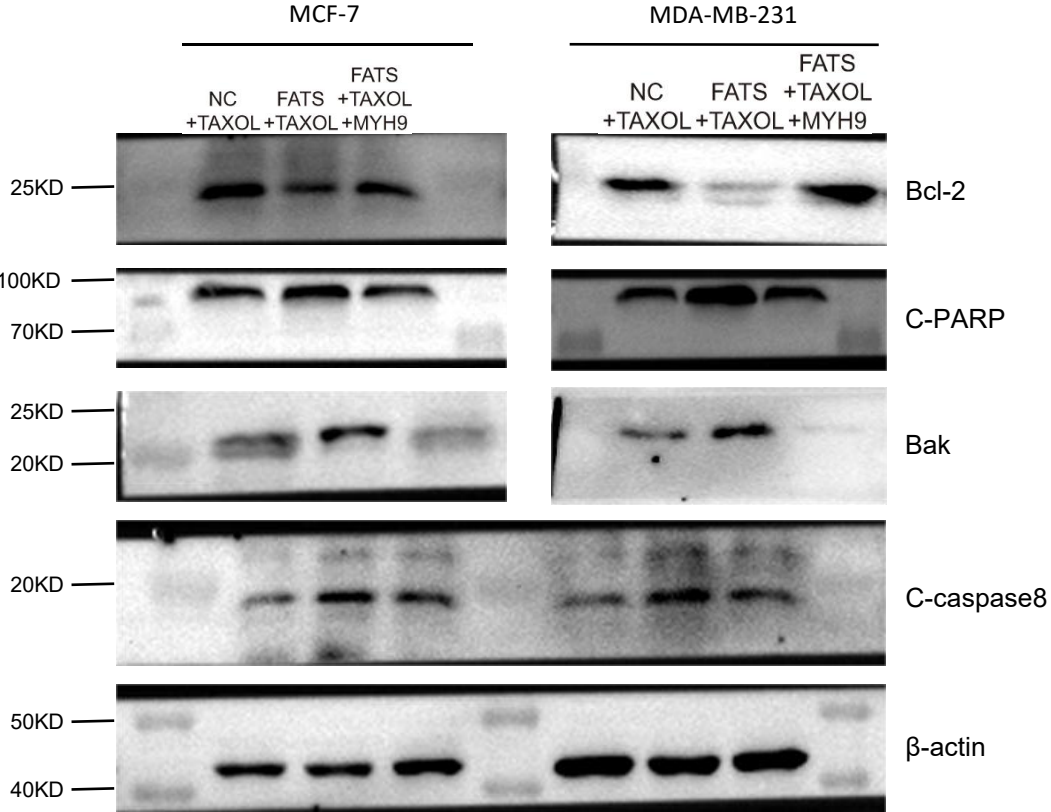

FigS1

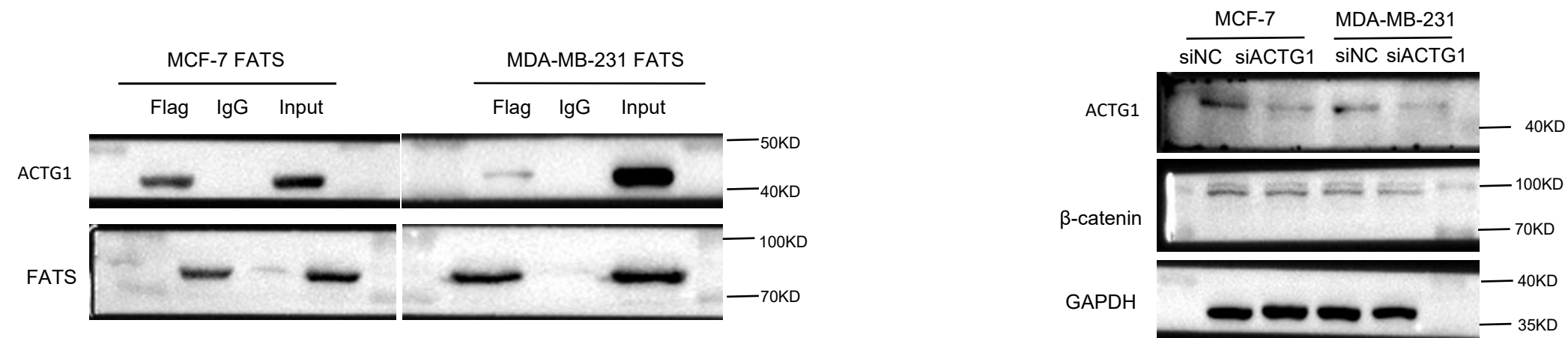

FigS2

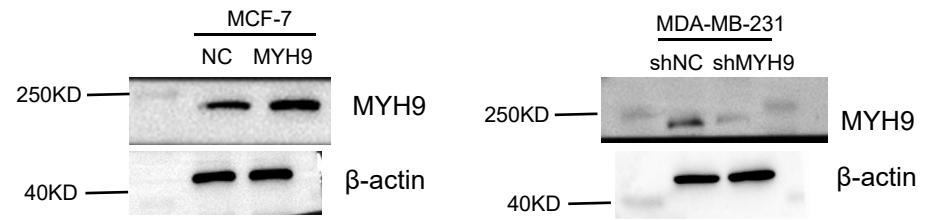

Supplement: Supplementary file 2 — western blot raw data [file 41419_2024_7164_MOESM2_ESM.pdf]
